# Structural Design Method for Constructions: Simulation, Manufacturing and Experiment

**DOI:** 10.3390/ma14206064

**Published:** 2021-10-14

**Authors:** Pavel Bolshakov, Nikita Kharin, Ramil Kashapov, Oskar Sachenkov

**Affiliations:** 1 Department Machines Science and Engineering Graphics, Tupolev Kazan National Research Technical University, 420111 Kazan, Russia; bolshakov-pavel@inbox.ru; 2 Institute of Mathematics and Mechanics, Kazan Federal University, 420008 Kazan, Russia; nik1314@mail.ru; 3 Institute of Engineering, Kazan Federal University, 420008 Kazan, Russia; kashramil.88@mail.ru

**Keywords:** structural design, porous constructions, additive manufacturing, CT

## Abstract

The development of additive manufacturing technology leads to new concepts for design implants and prostheses. The necessity of such approaches is fueled by patient-oriented medicine. Such a concept involves a new way of understanding material and includes complex structural geometry, lattice constructions, and metamaterials. This leads to new design concepts. In the article, the structural design method is presented. The general approach is based on the separation of the micro- and macro-mechanical parameters. For this purpose, the investigated region as a complex of the basic cells was considered. Each basic cell can be described by a parameters vector. An initializing vector was introduced to control the changes in the parameters vector. Changing the parameters vector according to the stress-strain state and the initializing vector leads to changes in the basic cells and consequently to changes in the microarchitecture. A medium with a spheroidal pore was considered as a basic cell. Porosity and ellipticity were used for the parameters vector. The initializing vector was initialized and depended on maximum von Mises stress. A sample was designed according to the proposed method. Then, solid and structurally designed samples were produced by additive manufacturing technology. The samples were scanned by computer tomography and then tested by structural loads. The results and analyses were presented.

## 1. Introduction

The modern approach for design implants and prostheses implies patient-oriented solutions. Such an approach involves not only new manufacturing methods but also a new vision of the product. Additive manufacturing allows production constructions with complex geometry. However, the solution for the automation of the design of such products is still open. So, nowadays lattice constructions have become popular for this purpose. Yet, the dependence between the different geometries of the lattice, the mechanical properties, and the biological adaptive is being researched [1,2,3]. Additionally, a manifestation of the brittle properties and the geometry deviations after manufacturing is still an issue of the day [4,5,6,7]. By changing the materials and melting modes [8,9,10], the mechanical parameters can be improved or vice versa. Despite the aforementioned difficulties, it is obvious that additive manufacturing and patient-oriented design can notably increase the quality of the medical treatments.

This article is focused on an approach for the structural design method. Previously, a method for designing a lattice endoprosthesis for long bones was developed [11]. The endoprosthesis was manufactured and passed clinical experiments. The developed approach was generalized. The main idea is based on the bone adaptation analogy. It is known that adaptation can be formulated by Wolff’s law [12]. To describe bone tissue orthotropy, a fabric tensor is used. The fabric tensor is also used to calculate the stiffness tensor [12,13]. The foundation of the adaptation model is an alignment of the stress and stiffness tensors. In terms of the fabric tensor, it means that the orthotropic directions are equal to the stress principal directions [13,14]. The widespread approach is to use representative volumes to determine the fabric tensor and the effective mechanical properties [15,16].

It has been shown [17,18,19] that implants interact with bone tissue and that the structure and the microstructure of the implant influence the quality of this interaction. Additive manufacturing allows the generation of solid irregular or lattice geometry [20,21], but on the other hand, local microporosity decreases fatigue resistance. Classical post-processing, such as tempering, allows the counteraction of the negative sides of the technology [22,23]. Despite the aforementioned technological barriers, the opening opportunities are promising. The ability to design material within a product opens up new possibilities in patient-specific prostheses [24,25]. The complexity of such an approach appears in defining the external loads and the formulation criteria of the design [26,27]. A novel approach is the use of additive manufacturing technology for liquid crystal elastomers, the exceptional properties of which show good usage in a range of applications in the fields of biology and medicine [28,29,30].

In this article, a method of structural design is presented. An example of structurally designed construction is presented. The designed and regular constructions were manufactured and compared in natural experiments.

## 2. Materials and Methods

### 2.1. Problem Formulation

**Figure 1:**
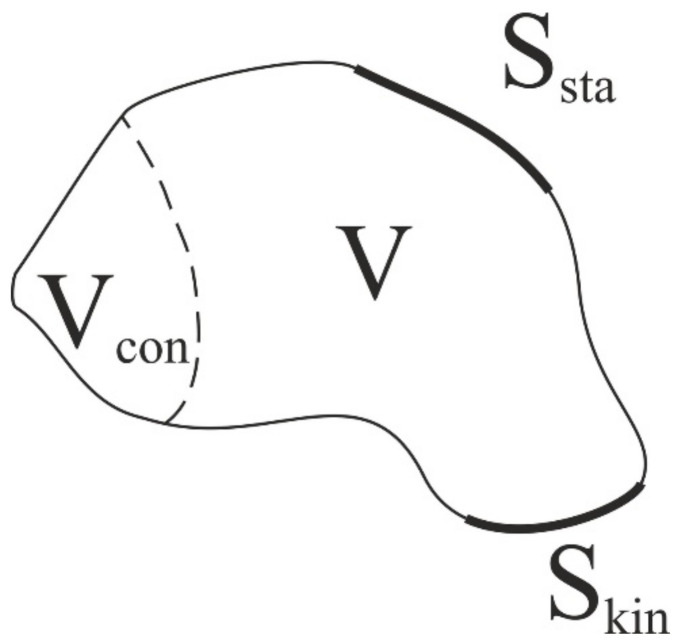
Scheme for problem formulation.

The mechanical behavior of the region *V* in *R*^3^ with the boundary *∂V*, within the linear theory of elasticity, can be described by the following system of equations [11]:

(1) $$\nabla \cdot \tilde{\sigma }=0,\;\forall \vec{x}\in V^{0}$$

(2) $$\tilde{\varepsilon }=\frac{1}{2}\left(\nabla \vec{u}+{\left(\nabla \vec{u}\right)}^{T}\right),\;\forall \vec{x}\in V^{0}$$

(3) $$\tilde{\sigma }=\overset{\tilde{˜}}{C}:\tilde{\varepsilon },\;\forall \vec{x}\in V^{0}$$

(4) $$\vec{u}=0,\;\forall \vec{x}\in S_{kin}$$

(5) $$\tilde{\sigma }\cdot \vec{n}=\vec{p},\;\forall \vec{x}\in S_{sta}$$

(6) $$S_{sta}\cup S_{kin}=\partial V$$

where *V°* = *V* ∪ *∂V*; *u* is the displacement vector; *σ* is the stress tensor; *ε* is the elastic strain tensor; and *C* is the stiffness tensor. *S_sta_* is the surface on which static boundary conditions are specified, and *S_kin_* is the surface on which kinematic boundary conditions are specified (see Figure 1).

It is necessary to find a distribution of the stiffness tensor *C* in the volume *V* such that the stress invariant (in our case, the von Mises stress) reaches a minimum at the constant boundary conditions.

(7) $$\overset{\tilde{˜}}{C}=\overset{\tilde{˜}}{C}(\vec{x}),\;\underset{\vec{x}\in V^{\prime }}{\max}\left\|\tilde{\sigma }\right\|\to \min$$

Applying the design conditions, it is necessary to determine the region *V_con_*, in which the components of the tensor of elastic properties remain unchanged:

(8) $$V_{con}\in V^{0}$$

Let us call the region *V_con_* the constant region. So, *V*′ in (7) can be determined as *V°\V_con_*. Adding Equation (7) to Equations (1)–(6) allows the formulating of the optimization problem for the structure.

### 2.2. Structural Problem Formulation

The general idea of the method is that the stress-strain state depends on some of the parameters vectors. Assuming that the anisotropy of the material is provided by the microarchitecture, we consider the forming material isotropic [13,16]. The parameters vector *λ* describes the material microarchitecture and influences the macro-stiffness tensor. On the other side, we should add an additional vector with initializing parameters, which describe the stress-strain state of the microarchitecture. Let us call it the initializing vector *γ*, which, obviously, depends on the invariants of the stress tensor *f*. The initializing vector can be interpreted as the control function of the microarchitecture changes. So, we propose that the stiffness tensor can be presented as a function of the parameters vector, the initializing vector, and the spatial coordinate:

(9) $$\overset{\tilde{˜}}{C}=\overset{\tilde{˜}}{C}\left(\vec{\lambda }\left(\gamma ,\vec{x}\right),\vec{\gamma }\left(f\left(\tilde{\sigma }\right),\vec{x}\right),\vec{x}\right)$$

Let us consider region *V* as the number of basic cells. For each basic cell we assume:

(10) $$\left\{\begin{array}{l} \vec{\lambda }\left(\gamma ,\vec{x}\right)=\vec{\lambda }\left(\gamma \right)\\ \vec{\gamma }\left(f\left(\tilde{\sigma }\right),\vec{x}\right)=\vec{\gamma }\left(f\left(\tilde{\sigma }\right)\right) \end{array}\right.$$

This approach considers a basic cell as a micro-construction with constant macro-properties. The parameters vector *λ* should be changed according to values of the initializing vector *γ*. So, if we introduce the control function *U* the problem can be rewritten:

(11) $$\left\{\begin{array}{l} \vec{\lambda }\left(\gamma ,\vec{x}\right)=\vec{\lambda }\left(\gamma ,U\left(\vec{x}\right)\right)\\ U\left(\vec{x}\right)=f\left(\vec{\gamma }\left(f\left(\tilde{\sigma }\right),\vec{x}\right)\right)\\ \overset{\tilde{˜}}{C}=\overset{\tilde{˜}}{C}\left(\vec{\lambda }\left(\gamma ,U\left(\vec{x}\right)\right),\vec{x}\right) \end{array}\right.$$

This means that the state of the initializing vector *γ* determines the changes of microarchitecture in terms of the parameters vector *λ*, and the microarchitecture influences the macro-stiffness tensor. Let us consider the investigated region as a composition of basic cells; each one describes the microarchitecture of a material. Each basic cell can be described by the parameters vectors and can be changed according to the initializing vector. To implement such an approach, the basic cell should be determined in order to define the parameters vector and its relationship with the stiffness tensor.

### 2.3. Basic Cell

In the research unit, a cube with a spheroidal pore was used as a basic cell. In this case, the parameters vector consists of porosity (*λ*) and the ellipticity coefficient (*β*). To investigate the dependence between the stiffness tensor and the parameters vector, a representative elements method was used [31,32,33]. For this purpose, the parameterized finite element model of a cube with a spheroidal pore was implemented. Twenty-node hexahedral finite elements were used. Kinematic loading was used in the numerical simulation. Uniaxial and shear loads in three directions were implemented. To clarify the mechanical properties, additionally combined (uniaxial with shear) loads were implemented [16,26,34,35]. The parameters were investigated in the interval (0; 1). According to the received data, the functions describing the influence of the parameters on the mechanical properties were found:

(12) $$\left\{\begin{array}{l} \vec{\lambda }=\vec{\lambda }\left(\lambda ,\beta \right)\\ E_{ii}=E_{ii}\left(\vec{\lambda }\right)\equiv C_{iiii}\left(\vec{\lambda }\right)\\ G_{ij}=G_{ij}\left(\vec{\lambda }\right)\equiv C_{ijij}\left(\vec{\lambda }\right)\\ \nu_{ij}=\nu_{ij}\left(\vec{\lambda }\right)\equiv C_{iijj}\left(\vec{\lambda }\right) \end{array}\right.$$

For approximation, a fourth-degree polynomial function was used with an approximation error threshold of about 0.9 [33,34,35,36]. In the calculations, some of the coefficients were equal to zero, so a common form of the final calculated polynomial was as follows:

(13) $$C_{ijkl}\left(\lambda ,\beta \right)=c_{00}+c_{10}\lambda +c_{01}\beta +c_{11}\lambda \beta +c_{21}\lambda^{2}\beta +c_{31}\lambda^{3}\beta +c_{12}\lambda \beta^{2}+c_{22}\lambda^{2}\beta^{2}+c_{13}\lambda \beta^{3}$$

where *λ* and *β* are components of the parameters vector—porosity and ellipticity, respectively, *c_ij_* are coefficients of the polynomial, where *i* shows the power of porosity and *j* shows the power of ellipticity. The received values of the coefficients for the approximation polynomial are listed in Table 1.

It should be noted that the polynomial coefficients for Poisson’s ratio can be reduced up to *c*_00_ because the influence of the parameters vector is insignificant. So, *ν*_12,13_ ≈ 0.011 and *ν*_23_ ≈ 0.017.

### 2.4. Proposed Algorithm

After the principal stress and directions are found, the orthotropic directions can be oriented according to the principal directions. The semi-major axis is directed to the 1st principal stress direction. The porosity is determined by von Mises stress and value [*σ*]_inf_. The [*σ*]_inf_ is the infimum of the stress value and determines the value of the underload. So, porosity can be restored by the equation:

(14) $$\lambda \left(\vec{x}\right)=\left\{\begin{array}{l} 1-\frac{{\left[\sigma \right]}_{\inf}-\sigma_{V.M.}\left(\vec{x}\right)}{{\left[\sigma \right]}_{\inf}},\sigma_{V.M.}\left(\vec{x}\right)<{\left[\sigma \right]}_{\inf}\\ 1,\sigma_{V.M.}\left(\vec{x}\right)\geq {\left[\sigma \right]}_{\inf} \end{array}\right.$$

To determine the ellipticity coefficient, the 1st and the 3rd principal stresses were used:

(15) $$\beta \left(\vec{x}\right)=\frac{\min\left(\left|\sigma_{1}\left(\vec{x}\right)\right|,\left|\sigma_{3}\left(\vec{x}\right)\right|\right)}{\max\left(\left|\sigma_{1}\left(\vec{x}\right)\right|,\left|\sigma_{3}\left(\vec{x}\right)\right|\right)}$$

Then, the stiffness constants can be calculated by porosity and the ellipticity coefficient and the stress-state problem can be solved. So, the algorithm can be described:
**Algorithm of structural design**1. Load a *mesh* and apply *boundary conditions*.2. Highlight *elements* from the *constant region* (8)3. Set initial parameters vector4. Solve the stress-state problem (1)–(6)5. **for** each *element* not from *constant region*6. Calculate the principal stress and directions7. Calculate the parameters vector (14), (15)8. Calculate stiffness tensor (13)9. Orient element coordinate system according to the principal directions.10. **end for**11. **if** not *stop*
**goto** 412. Restore geometry by parameters vector.

### 2.5. Model Task

**Figure 2:**
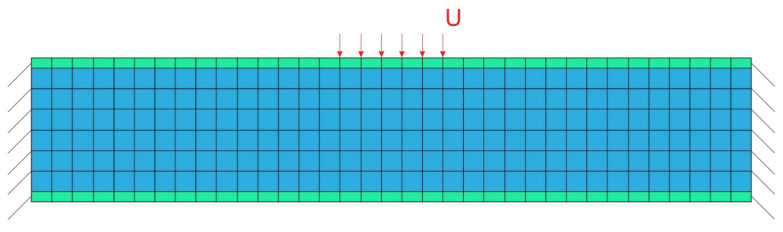
Loading scheme; *U* is applied displacements; the green region is *V_con_* region.

A rectangular beam of 140 mm × 28 mm × 14 mm was used for the algorithm implementation. Eight-node hexahedral finite elements were used for the calculations. The kinematic loading of 1 mm was used in the numerical simulation. The length of the kinematic loading region was 20 mm. In Figure 2, the loading scheme is presented; the *V_con_* region is marked by a green color. The end faces of the beam were fixed.

The mechanical properties of acrylonitrile butadiene styrene were used for further production by additive manufacturing. So, Young’s modulus was equal to 200 GPa, the shear modulus was equal to 71.5 GPa, and the Poisson ratio was 0.4. For [*σ*]_inf_, 10% of maximum von Mises stress in the construction was used. The stop condition was as follows:

(16) $$\max\left(\left|\lambda_{i}-\lambda_{i-\prime }\right|,\left|\beta_{i}-\beta_{i-1}\right|\right)<\varepsilon$$

where *ε* was equal to 10^−3^.

### 2.6. Experiments

After restoring the geometry, the beam was produced by additive manufacturing technology. Acrylonitrile butadiene styrene was used for the manufacturing. Both the solid and the structural design samples were produced. For every two types of samples, longitudinal and transverse directions of printing were used. Computed tomography (CT) (Vatech PaX-I 3D, Kazan, Russia) was used to estimate the structure. After that, three-point bending was carried out and stress-strain curves were obtained for all samples. For the stress-strain curves, the ultimate force and slope were analyzed.

## 3. Results and Discussion

**Figure 3:**
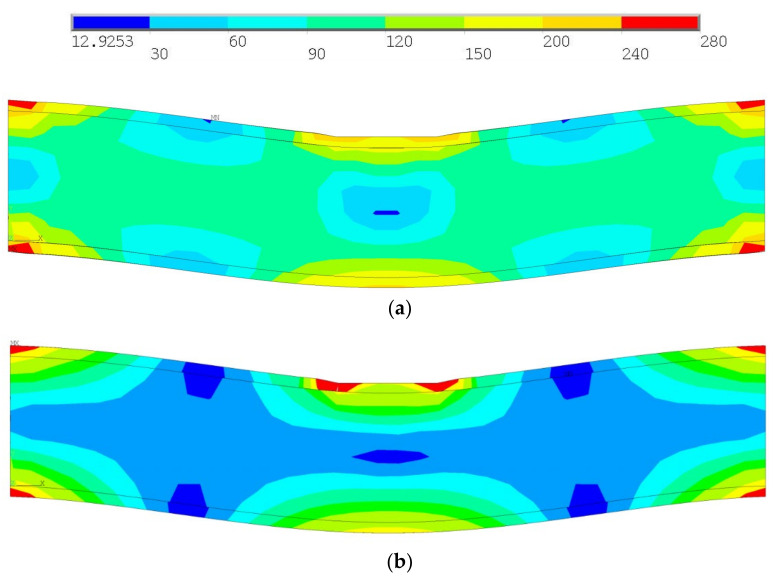
Von Mises stress distribution for initial (**a**) and structurally designed (**b**) beam.

The stress-strain state for the initial (solid) and the structurally designed beam were compared. The maximum stress did not change significantly, but the distribution of stress inside the product decreased (see Figure 3). The algorithm showed fast convergence; it was about 38 iterations. The maximum stress was localized in the zones of kinematic constraints for the initial geometry. In addition, for the structurally designed beam the maximum stress was localized in the zones of kinematic boundary conditions (see red regions in Figure 3). On the other hand, zones of stress reduction appeared for the structurally designed beam (see blue regions in Figure 3).

**Figure 4:**
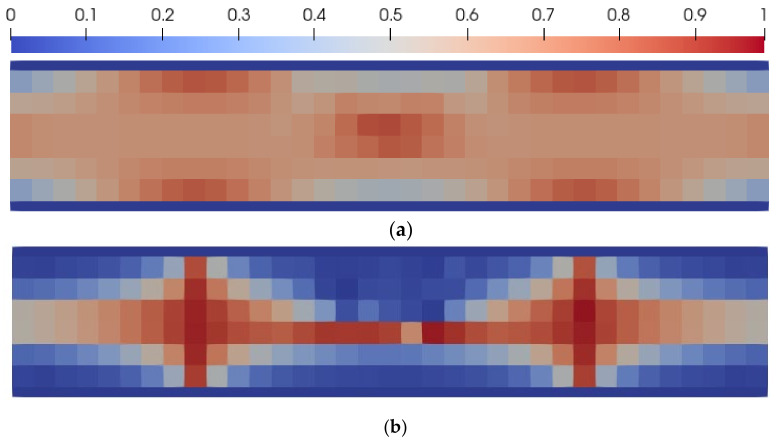
Distribution of porosity(**a**) and ellipticity coefficient (**b**).

The distribution of the received porosity and ellipticity coefficients is shown in Figure 4. The zones of high porosity are localized where the von Misses stress was minimal (red zones in Figure 4a). In the same zones, the pore’s ellipticity coefficient is close to 1 (red zones in Figure 4a), which means that in this region the pore is almost spherical.

**Figure 5:**
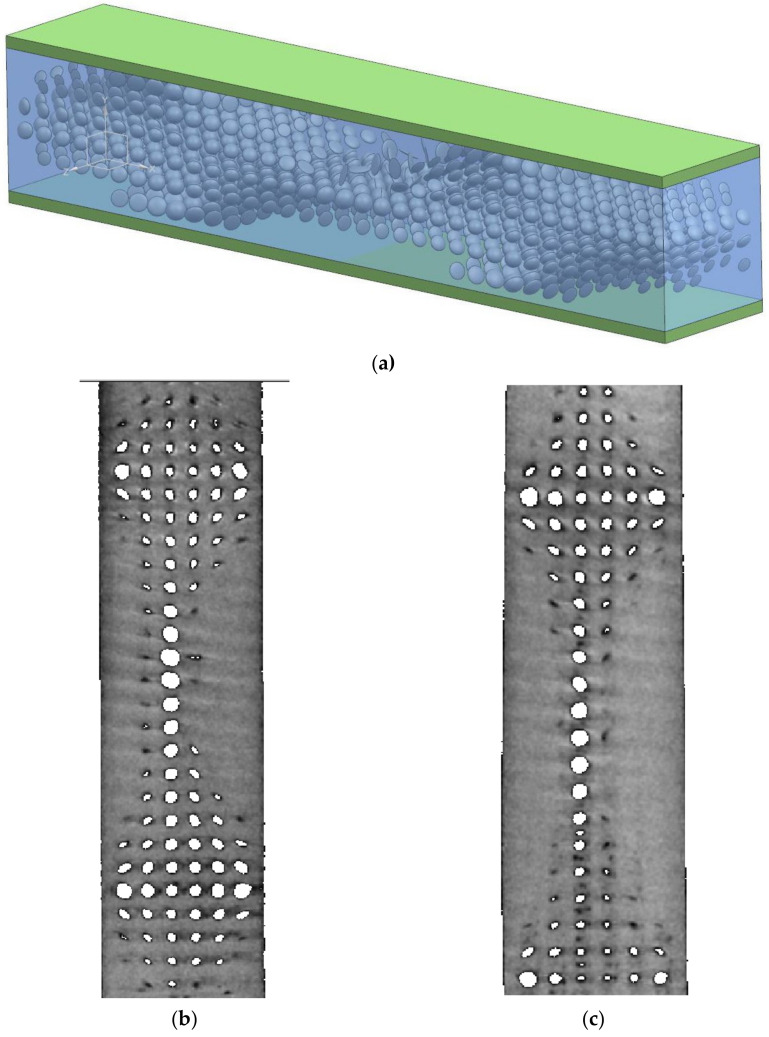
Restored 3D geometry (**a**), CT scans for longitudinal printing (**b**), and transversal printing (**c**).

The 3D geometry was restored for the following manufacturing (see Figure 5a). The initial and structurally designed samples were manufactured in two ways: longitudinal and transversal printing. After the manufacturing, the samples were scanned by CT (see Figure 5b,c). The deviations of pore geometry in the manufactured samples were noted. They were caused by the cooldown speed of the printing material drop. However, the distribution of the porosity was close enough to the design (deviations about 5%).

**Figure 6:**
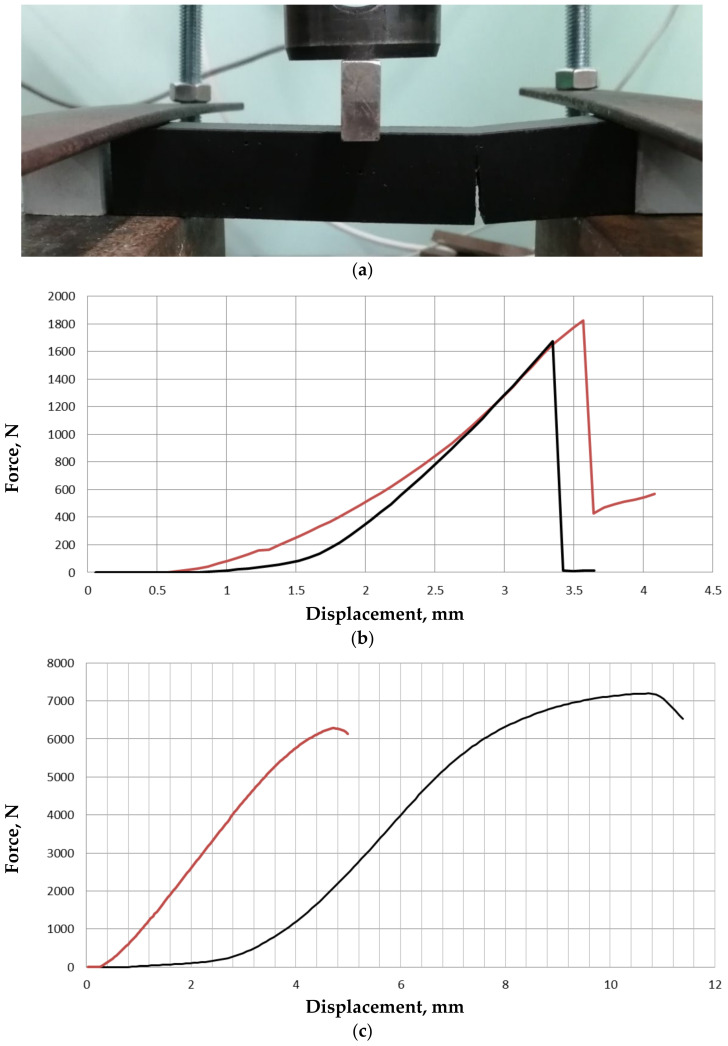
Three-point bending scheme (**a**), a stress-strain curve for longitudinal printing (**b**), and transversal printing (**c**); black lines—initial geometry, red lines—structurally designed geometry.

In the three-point bending experiments for the initial geometry, which was longitudinally printed, the maximum force was 1675 N, and the maximum displacement was 3.35 mm. For the structurally designed geometry, which was longitudinally printed, the maximum force was 1825 N, and the maximum displacement was 3.56 mm. A crack appeared in the middle, in the longitudinal direction between the kinematics constraints and the applied force (see Figure 6a). In the three-point bending experiments for the initial geometry, which was transversally printed, the maximum force was 7196 N, and the maximum displacement was 10.73 mm. For the structurally designed geometry, which was transversally printed, the maximum force was 6271 N, and the maximum displacement was 4.76 mm. A crack appeared under the applied force. The stress-strain curves for all the cases are shown in Figure 6b,c. The ultimate force deviation for the initial and structurally designed cases was about 10%, and it could be decreased by improving the manufacturing of the samples. A significant difference was noted for the displacements in the case of the transversal printing. The structurally designed sample became more rigid (4.76 mm vs. 10.73 mm).

Comparing the slope (for the longitudinal printing), a 25% increase was noted for the structurally designed sample (1184 N/mm and 1491 N/mm, respectively). The slope in the case of the transversal printing decreased by 20% for the structurally designed sample (710 N/mm and 894 N/mm, respectively).

## 4. Conclusions

The algorithm for the structural design of the geometry was proposed. In the framework of the study, a porous cube was chosen for the basic cell. The following assumptions were used: calculation was carried out in an elastic zone; the material is isotropic; and anisotropy appears by the porosity of the basic cell. The iterative algorithm for the structural design was presented. The samples were designed, and the verification of the structural simulations was carried out. The comparison of the maximum von Mises stress for all the samples did not show a significant difference. However, for the structurally designed beam, zones of stress reduction appeared.

The manufacturing was provided using additive technologies. The samples were printed using different directions, and three-point bending tests were performed. Stress-strain curves were obtained for all the samples. In the case of the longitudinal direction printing, the ultimate force of the structurally designed sample was about 10% higher. In the case of the transversal direction printing, the rigidness of the structurally designed sample was almost 40% higher. The analysis of the stress-strain curves for all the samples shows the significant influence of the printing directions on the mechanical properties and demonstrated the need for post-processing.

## Figures and Tables

**Table 1:** The values of coefficients of approximation polynomial for stiffness parameters.

	*c*_00_	*c*_10_	*c*_01_	*c*_11_	*c*_21_	*c*_31_	*c*_12_	*c*_22_	*c*_13_
***E*_11_**, GPa	109	−3.9	−5.3	−192	287	−115	319	−209	−136
***E*_22,33_**, GPa	102	2.9	10.6	−111	325	−278	−17.8	−18.7	27
***G*_12,13_**, GPa	10.7	−0.1	0.25	−2.7	13	−10	−3.9	−0.1	4.1
***G*_23_**, GPa	2.5	−0.1	−0.06	−4.4	8	−3.4	6.4	−5	−2.5
***ν*_12,13_**	0.011	−0.005	−0.009	−0.032	−0.038	0	−0.027	0.464	0
***ν*_23_**	0.017	−0.049	−0.017	−0.07	0.4	0	0.09	−0.18	0

## Data Availability

Data available on request.
